# Cannabis use experience of patients with chronic disease after revisions to the cannabis legalization regulations: a mixed-methods study in primary care settings in the south of Thailand

**DOI:** 10.1017/S146342362510056X

**Published:** 2025-11-03

**Authors:** Supakorn Sripaew, Phoomjai Sornsenee, Polathep Vichitkunakorn, Sawitri Assanangkornchai, Orapan Fumaneeshoat

**Affiliations:** 1 Department of Family and Preventive Medicine, Faculty of Medicine, Prince of Songkla University, 15 Kanchanavanich road, Hat Yai district, Songkhla province 90110, Thailand; 2 Department of Epidemiology, Faculty of Medicine, Prince of Songkla University, 15 Kanchanavanich road, Hat Yai district, Songkhla province 90110, Thailand

**Keywords:** Cannabis, chronic disease, complementary and alternative medicine, health policy, primary care, self-management

## Abstract

**Aim:**

To understand patterns of cannabis use and self-management experiences in patients with chronic disease during the post-legalization period in Thailand and to quantify such experiences and perceptions.

**Background:**

Patients with chronic disease are a population in which disease self-management is potentially involved with the use of complementary and alternative medicines (CAMs). The recent changes in cannabis regulation in Thailand have allowed retail selling and home cultivation. Cannabis is a medicinal herb in many traditional Thai recipes and is often adopted as a CAM in the chronic disease population. The adoption of cannabis products as part of CAM could lead to changes in chronic disease outcomes.

**Methods:**

Exploratory-sequential mixed methods were used in this study. A descriptive qualitative study was conducted to acquire a basic understanding of the patients’ experiences. Semi-structured in-depth interviews were conducted, and thematic analysis was applied. Subsequently, a cross-sectional study was conducted to quantify the patterns of cannabis use and self-management experience in patients with diabetes and/or hypertension.

**Findings:**

Eleven patients were interviewed, and 124 patients participated in the cross-sectional study. Most of the participants were male, married, and identified as Buddhist. Many patients believed that cannabis could improve their health, while fewer considered it a threat to their health. In general, the patients viewed cannabis as a way to enhance their quality of life and treat chronic diseases. Some patients embraced the principles of CAM. They primarily used cannabis tea daily to manage diabetes or hypertension, with their approaches being more complementary than alternative. However, only one-third (34.7%) were aware of potential drug interactions with their concurrent medications.

## Introduction

The umbrella term ‘chronic disease’ refers to diseases that have a long period of development, have a prolonged course, and are associated with long-term functional disabilities, such as cardiovascular diseases (CVDs), cancers, chronic respiratory diseases, diabetes, hypertension, and hypercholesterolemia (Wilper *et al.*, 2008; Bernell and Howard, 2016; *Non communicable diseases*, 2023). Globally, a recent Organization for Economic Cooperation and Development (OECD) study reported that the prevalence of chronic conditions was approximately 27% and 43% in the lowest- and highest-income quintile populations, respectively (OECD, 2021). The increasing incidence of chronic diseases in Thailand is a major public health concern. The 2022 National Health Examination Survey (NHES VI) (Aekplakorn, 2021) found that 0.4, 1.5, 9.5, 25.4, and 53.8% of the working-age population had chronic respiratory diseases, CVDs, diabetes, hypertension, and hypercholesterolemia, respectively, with these rates being higher than those in a previous 2013 report (Aekplakorn, 2013).

Patients with chronic diseases often focus on self-management of their condition. A general chronic disease care framework encourages the health system and community to support chronic disease self-management (Wagner *et al.*, 2001), which covers three areas: (1) specific disease management (e.g., medication or diet adherence), (2) lifestyle modifications (e.g., altering routine behaviors), and (3) emotional impact management (e.g., coping with stress) (Corbin and Strauss, 1985; Lorig and Holman, 2003). Studies have found that people with chronic disease exhibit various health-related self-seeking behaviors involving complementary and/or alternative medicines (CAM), such as vitamins, herbs, and commercial products, which can affect medication adherence and exercising (Bell *et al.*, 2006; Vidot, Lerner, and Gonzalez, 2017; Rajahthurai *et al.*, 2022).

Cannabis is a genus of medicinal, recreational, and fibrous plants that contain hundreds of compounds, from which cannabinoids are processed in various ways and sold. There are several licensed and unlicensed cannabis products, such as dried cannabis, edibles, extracts, and synthetic cannabinoids (Freeman *et al.*, 2019). Many cannabis products are popular CAM options. Studies have reported that 37.2, 50.2, 4.2, and 38.3% of patients with cancer, chronic kidney disease, Parkinson’s disease, and Human Immunodeficiency Virus (HIV), respectively, had used cannabis at some time during their illness as a CAM. Medical purposes were the major reason for patients with cancer, Parkinson’s disease, HIV, or other diseases, whereas patients with chronic kidney disease commonly used cannabis recreationally (Finseth *et al.*, 2015; Vidot, Lerner, and Gonzalez, 2017; Collister *et al.*, 2022; Vinette *et al.*, 2022).

Cannabis regulations vary globally, with medicinal and recreational use regulations not uniform across countries (Areesantichai, Perngparn and Pilley, 2020). Unlike many Asian countries, such as Indonesia and Malaysia, which criminalize all cannabis activities (Yustina *et al.*, 2023), Thailand relaxed its cannabis laws in 2019, 40 years after the Narcotic Acts had made cannabis illegal. Initially, cannabis exploitation for research and medical prescriptions was legalized. In June 2022, Thai regulations extended cannabis use by removing cannabis plants with a delta-9 tetrahydrocannabinol (THC) concentration ≤0.2 mg percent from the prohibited narcotics list, allowing individual possession and home cultivation while prohibiting retail sales to minors (under 20 years of age) and pregnant women. However, aspects of the revised cannabis control measures remain unclear, and amendments to clarify these aspects are being debated (Sornpaisarn *et al.*, 2023). Following the legalization of medical cannabis in 2019, a study found that 24.5% of patients with chronic diseases used cannabis for medical purposes (Assanangkornchai *et al.*, 2022), with a slight increase in the consumption of illegal edible cannabis products (Kalayasiri and Boonthae, 2023).

Thai people have recognized cannabis as a medicinal herb for centuries, and it is used in 16 traditional medicine recipes and certain dishes (*Pharmaceutical manual for cannabis recipe in the Thai traditional medicine for public healthcare centers*, 2023). The people of southern Thailand have several traditional recipes that include various medicinal and narcotic herbs, including cannabis (used for hypnosis) (Chuakul *et al.*, 2004) and kratom (*Mitragyna speciosa*), which is very common in the south (Charoenratana, Anukul, and Aramrattana, 2021). However, since the significant changes to the narcotics laws in June 2022, public health authorities, especially primary care workers, do not have sufficient information regarding the cannabis use patterns of patients with chronic disease or any alterations in their disease management related to cannabis use. Thus, they lack awareness of possible cannabis use behaviors that could affect chronic disease care.

Due to the potential impact of cannabis use on the care of chronic disease, it is crucial for primary care stakeholders to be informed about potential cannabis adoption in those with chronic disease. Therefore, the current study aimed to understand the disease self-management experiences and perceptions of patients with chronic disease who consumed cannabis during the post-legalization period and quantitatively describe such experiences, perceptions, and patterns of cannabis use in this population in the south of Thailand.

## Methods

### Study design and participants

This study used an exploratory sequential mixed-methods approach. The first phase of the study was a descriptive qualitative study using thematic analysis of patient interview scripts to explore cannabis use patterns among patients with chronic disease in Songkhla Province, Thailand. At the time when the study was conceptualized, the knowledge of cannabis use behaviors, awareness regarding cannabis, and perceptions among those with chronic disease had never been explored. Thus, our qualitative study allowed the questionnaire to be applied later in the second phase to query contextually relevant information (i.e., identifying item contents to be measured) from the target population. The second phase was a cross-sectional study designed to further quantify cannabis knowledge, perceptions, and behaviors among patients with chronic disease in the province through questionnaire completions. The first phase was conducted between February 1, 2023, and June 30, 2023, and the second phase took place between July 1, 2023, and January 31, 2024.

### Setting

Data collection for both phases was conducted at four non-communicable-disease (NCD) clinics representing the four levels of government hospitals in Thailand: (1) community hospitals (CH), (2) general or provincial hospitals (GH), (3) regional hospitals (RH), and (4) medical school hospitals (MH). Within the overall health system, the most common diseases are diabetes and hypertension, which are primarily managed by NCD clinics operating in primary care settings.

### Data collection

#### Phase 1: qualitative study

The first phase involved a qualitative study, semi-structured in-depth interviews, and retrospective reviews of secondary data (e.g., government medical and Thai traditional cannabis clinic reports). We selected primary care patients aged ≥35 years who had NCDs, that is, diabetes, hypertension, cardiovascular diseases, chronic lung disease, and/or any type of neoplasm, who voluntarily disclosed their use of cannabis products to healthcare providers at NCD clinics in Songkhla province. Additionally, we interviewed persons involved with chronic disease self-management and cannabis product consumption, including family members, physicians, nurses, and local cannabis store owners, to obtain deeper insights and establish data triangulation where possible. We defined cannabis products as any type of product containing cannabinoids (legal or illegal) or any part of the cannabis plant. The authors (SS and PS) discussed the interview framework before beginning the interviews and developed an interview guide. The semi-structured interview was piloted with two informants. Prior to each actual interview, the interviewer had never seen the informant, and their attitudes toward cannabis use were neutral. The interviews were audio-recorded, and non-verbal communications were recorded using field notes. Each interview lasted for approximately one hour. The audio tapes were transcribed verbatim. When the informant was able and willing to provide a sample of their cannabis product, it was sent for cannabidiol concentration tests (delta-9 THC and cannabidiol levels) to examine the composition of the plants/products. All homemade products were tested as received while cannabis drinks were pasteurized before testing (Hazekamp *et al.*, 2007).

#### Phase 2: cross-sectional study

For phase 2, we developed a multi-section questionnaire to further quantify the awareness, attitudes, and behaviors of patients with chronic disease to use in the cross-sectional study. We used stratified sampling to recruit different patients from phase 1 from four selected NCD clinics, one representing each level of government hospital (CH, GH, RH, and MH), of which the size of the sample from each clinic was proportional to the total NCD patients served in each setting. Given the predominance of diabetes and hypertension over other chronic diseases in our setting, the study population for the second phase focused on individuals with diabetes and hypertension. Eligible patients were those who had hypertension, diabetes, or both conditions; had used cannabis products in the past 3 months; and were aged ≥35 years. All those with diabetes and hypertension who visited the study sites during the second part of the study period were briefly informed about the study, with a printed sheet explaining the study details. The questionnaires were distributed to interested participants for self-completion, or a structured interview was conducted at the participant’s request. The participants’ prescriptions were examined for any drug-drug interactions with their consent. Before the data collection, we calculated the required sample size to estimate a 50% proportion of cannabis smoking and tea use in an infinite population of patients with chronic disease who used cannabis. At alpha = 0.05 and error margin = 0.1, the required sample size was 97 (Daniel, 1995).

### Research instruments

We developed a questionnaire based on the subthemes identified in our qualitative analysis. The knowledge questionnaire items were based on yes/no answers, while the perception questionnaire used a 5-point Likert scale (totally disagree, disagree, uncertain/neutral, agree, and totally agree). The content validity of the questionnaire was assessed by five experts (two epidemiologists, two family physicians, and one community medicine specialist), and the item-objective-congruence indexes for all items were higher than the minimal acceptable values (0.60/1.00). The items in the perception questionnaire demonstrated internal consistency, with an alpha coefficient of 0.78. The Thai version of the Alcohol, Smoking, and Substance Involvement Screening Test (ASSIST) was used to assess health risk levels associated with cannabis consumption (WHO ASSIST Working Group, 2002). Risk levels were categorized based on the ASSIST score as low (0–3 points), moderate (4–26 points), and high (≥27 points). Medication adherence was assessed using the Medication Adherence Scale for Thais (MAST) (Jongwilaikasem and Lerkiatbundit, 2021). This instrument comprises eight items that assess the frequency of performing various behaviors, representing specific treatment adherence features. The scores on this instrument ranged from never (0) to very frequently (5). A total adherence score ≥33 is considered good adherence. Additionally, the Thai Ministry of Health’s brief indicators for healthy behaviors (‘Tools for health literacy and healthy behavior assessment for adult age 15-59 years’, 2018) were used to categorize the frequency per week (never, 1–2, 3, 4–5, and 6–7 days) of six deemed healthy behaviors: (1) limiting calories, salt, sugar, and fat; (2) consuming at least half a kilogram of fruits and vegetables daily; (3) maintaining moderate physical activity; (4) dealing with problems using positive thoughts; (5) refraining from smoking cigarettes; and (6) refraining from consuming alcohol.

### Data analyses

Thematic analysis was conducted following previous guidelines (Braun and Clarke, 2006). SS and PS were responsible for coding the data. Initially, they familiarized themselves with the interview transcripts before beginning free coding using spreadsheets. The codes were organized into groups, and iterative modifications were applied to refine the initial codes. Themes emerged inductively from the data through this process. Accessible participants were contacted to provide feedback on the findings. Data from the cross-sectional study were analyzed using descriptive statistics. For the analysis of perceptions, we grouped ‘totally disagree’ with ‘disagree’ and ‘totally agree’ with ‘agree’ for simplicity. The characteristics of cannabis use are shown as point estimations of prevalence and 95% confidence intervals. An example of representative verbatim quotes is provided alongside the qualitative description. Microsoft Excel and R software (stringr, lubridate, ggplot2, and epicalc packages) were used for data management and analyses.

### Findings

Thirteen patients identified as cannabis users were invited to an interview. Two patients refused to participate due to legal concerns, as they were uncomfortable revealing that they had any associations with cannabis. Eleven patients, one family member, two local recreational cannabis shop owners, two nurses, one doctor, and one folk healer participated in the in-depth interviews. Fifty hundred and sixty patients visiting the study centers were informed of the study. One hundred and twenty-four patients using cannabis products participated in the cross-sectional study. Table 1 shows the informants’ characteristics (qualitative study) compared to participants from the sampled NCD clinics (cross-sectional study). In both phases, most participants were male, married, and Buddhist. The participants in the two studies differed in monthly family income, education, occupation, and health insurance scheme. Most (90.3%) of the NCD clinic patients had hypertension.

**Table 1.** Patient participants’ characteristics

Characteristic	Qualitative study informants (*n* = 11)	NCD clinic participants (*n* = 124)
Age in years (mean, SD)	62.1 (14.5)	61.9 (10.1)
Male [*n* (%)]	10 (90.9)	73 (58.9)
Religion [*n* (%)]		
Buddhist	7 (63.6)	103 (83.1)
Islam	4 (36.4)	20 (17.6)
Not reported	0 (0)	1 (0.8)
Marital status [*n* (%)]		
Married	11 (100)	107 (86.2)
Single	0 (0)	8 (6.5)
Widowed/divorced	0 (0)	9 (7.3)
Family monthly income [*n* (%)]		
800 USD or lower	2 (18.2)	112 (90.3)
801 – 1,200 USD	5 (45.5)	8 (6.5)
Higher than 1,200 USD	3 (27.3)	4 (3.2)
Unsure	1 (9.1)	0 (0)
Education [*n* (%)]		
Primary school or lower	2 (18.2)	71 (57.3)
High school or equivalent	5 (45.5)	43 (34.7)
Bachelor’s degree or equivalent	4 (36.4)	10 (8.1)
Occupation [*n* (%)]		
Self-employed (e.g., running convenience store)	3 (27.3)	28 (22.6)
Employee	3 (27.3)	23 (18.5)
Agriculture/fishing	2 (18.2)	8 (6.5)
Government or professional worker (e.g., lawyer, healthcare worker)	3 (27.3)	17 (13.7)
Housewife/unemployed	0 (0)	48 (38.7)
Health insurance scheme [*n* (%)]		
Universal health scheme	3 (27.3)	77 (62.1)
Social security scheme	1 (9.1)	13 (13.1)
Civil servant health scheme	4 (36.4)	30 (24.2)
Other health scheme	3 (27.3)	3 (2.4)
Had diabetes [*n* (%)]	5 (45.5)	78 (62.9)
Had hypertension [*n* (%)]	4 (36.3)	112 (90.3)
Had cancer/tumor [*n* (%)]	2 (18.2)	2 (1.6)
Had cardiovascular disease (e.g., coronary disease, stroke) [*n* (%)]	1 (9.1)	12 (9.7)
Perceived health status [*n* (%)]		
Very healthy	2 (18.2)	8 (6.5)
Healthy	3 (27.3)	37 (29.8)
Moderate	4 (36.4)	65 (52.4)
Poor	1 (9.1)	6 (4.8)
Very poor	1 (9.1)	8 (6.5)
Follow-up hospital [*n* (%)]		
Community hospital	3 (27.3)	21 (16.9)
General hospital	2 (18.1)	60 (48.4)
Regional hospital	4 (36.3)	31 (25.0)
Medical school hospital	2 (18.1)	12 (9.7)

### Theme 1: perceptions related to cannabis

**Figure 1.**
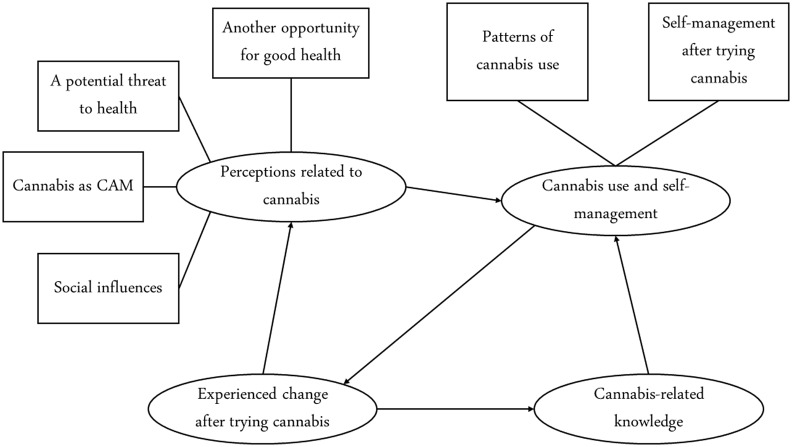
Thematic diagram of chronic disease self-management experiences among patients using cannabis products. Caption: one-headed arrows denote a unidirectional relationship between themes.

**Figure 2.**
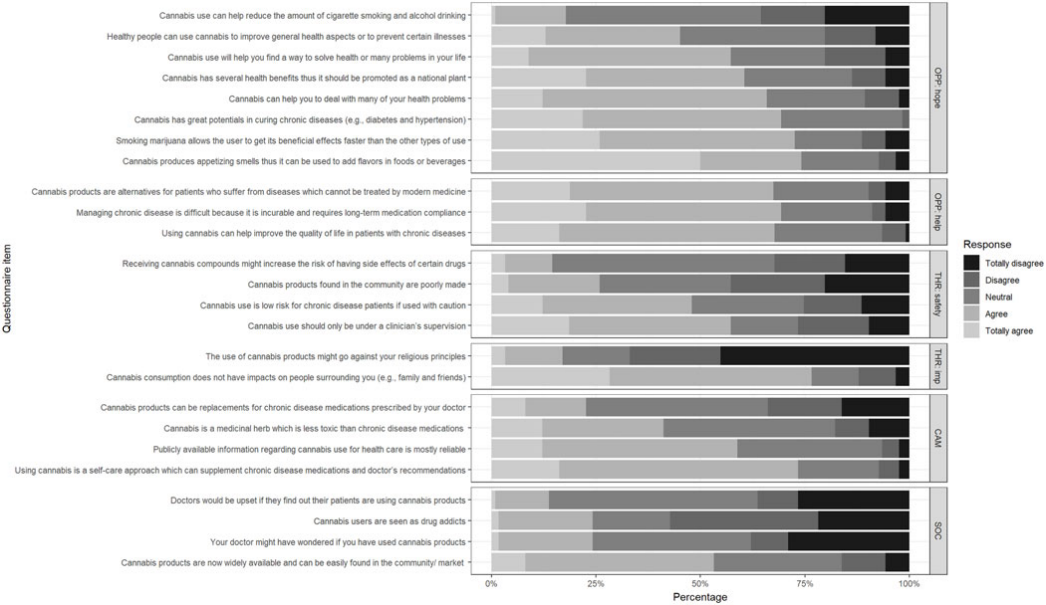
Perceptions regarding cannabis consumption in the NCD clinic sample. Caption: Uppercase and lowercase letters in the facet labels denote the subtheme and subcategory, respectively. Subtheme 1, ‘Cannabis is another opportunity for improving health’, has two subcategories: alternative hope for better health [OPP: hope] and helping reduce the burden of untreatable disease [OPP: help], Subtheme 2, ‘cannabis as a potential threat to health’, has two subcategories: safety of cannabis use [THR: safety] and impacts of cannabis on health aspects [THR: imp]. No subcategories were defined in subtheme 3, ‘Cannabis as complementary and alternative medicine’ [CAM], or subtheme 4, ‘Social influences’ [SOC].

The participants with chronic disease shared their perceptions related to cannabis products as an opportunity for improving health, a potential health threat, cannabis as CAM, and social influences. Figures 1 and 2 shows the perceptions of participants regarding the subthemes and other subcategories.

#### Subtheme 1: another opportunity for good health

*‘I was too worried to use it in the past. However, now, I have no concerns. I think its effects are too good to be considered harmful … it might help depressed persons. I think this cannabis … cures depression … if we smoke a roll of cannabis, it will direct our thoughts away from negativity’. (Male 65, retired lab technician, diabetes)*

More than half (60.5%) of the participants believed that cannabis was a promising commercial medicinal herb. Most (67.7%) saw cannabis as a new option that might improve their quality of life. The chronicity of symptoms, long-running treatments (burdensome for certain patients), and the incurable nature of chronic diseases were concerns of most users (69.4%). A small majority (65.3%) used cannabis to help them find solutions to some of their problems. Another special use pertained to the enhancement of spiritual activity. Some patients (17.7%) believed that cannabis could be used for cigarette and/or alcohol cessation support, and many (72.6%) believed there were faster beneficial effects from smoking cannabis than via the other ingestion routes (Figure 2) .

#### Subtheme 2: a potential threat to health

*‘During Ramadan, my hands shake frequently … I asked a diabetic person I knew what was going on; he said it could be somewhat from the (cannabis) tea … During my last Ramadan fasting, I think I must have been unable to handle it … so I stopped using it (cannabis tea). Since then, I have never experienced such exhaustion again’. (Male 52, fishery, diabetes)*

Although many (47.6%) participants felt cannabis was safe under certain precautions, 12 (14.5%) were aware that using cannabis with other medications could increase the risk of medication side effects. More than half (57.2%) thought that cannabis should only be used under the doctor’s supervision. Moreover, cannabis use was concerning for some participants because of their religious beliefs (16.9%).

#### Subtheme 3: Cannabis as CAM

*‘People say “doctors prescribe (anti-diabetes) medicines for their patients but do not prefer to use them for their diabetes management … We can first focus on controlling diabetes with the doctor’s medicines, then we should step down and try medicinal herbs”’. (Male 65, retired lab technician, diabetes)*

Our participants mainly considered cannabis products as supplements (73.4%) rather than replacements for chronic disease medication (22.6%). Many (41.1%) believed that cannabis was a way to manage the disease with less medication. More than half (58.9%) trusted the cannabis information obtained from general sources or from their acquaintances.

#### Subtheme 4: social influences

*‘Nowadays, there are people who understand (the commonness of cannabis use). However, some people are disgusted. They see us as addicts. One side sees cannabis as a type of medicine, but the other side always tries to take us down. I sometimes came across these people. But I felt like I was the one doing the wrong thing, so I left’. (Male 60, self-employment, fully recovered from stroke)*

Approximately a quarter (24.2%) of our participants thought that other people might have seen them as illicit drug users. A small number (13.7%) were concerned that there could be disagreements with their doctors because of discordant perceptions toward cannabis, although most (75.8%) thought that its use would not concern their doctors. More than half of the patients (53.3%) perceived that cannabis was easy to find. We additionally learned from the clinic staff that some patients had been lost to follow-up after a period of cannabis product supply shortage in several government hospitals during the pandemic (patient visits decreased from five to two patients/month in the year before the study).

### Theme 2: Cannabis-related knowledge

*‘We are lucky that (cannabis products) were legalized. Many patients had a higher chance of surviving their cancers after this change’. (Male 81, self-employment, advanced esophageal cancer)*

Some participants misunderstood the risks and benefits of cannabis and the current situation of cannabis regulation in Thailand. Only approximately one-third of the participants had a correct understanding of the regulations pertaining to cannabis use, medically proven cannabis use for cancer treatment, and potential cannabis-drug interactions (38.7, 31.5, and 34.7%, respectively). Table 2 shows the knowledge and understanding about cannabis among NCD clinic participants.

**Table 2.** Knowledge characteristics of chronic disease patients in the NCD clinics sample

Characteristic	% correct (95% CI)
Consuming different parts of cannabis plants produces different effects	90.3 (85.0 – 95.6)
High cannabis dosages could make the users confused or agitated	85.5 (79.2 – 91.8)
The use of cannabis would increase the risk of road accidents	83.1 (76.4 – 89.8)
The same cannabis strain could have different levels of active compounds	81.5 (74.5 – 88.4)
Standardized cannabis products can be used to increase appetite in some patients	81.5 (74.5 – 88.4)
Some cannabis products are addictive	78.2 (70.9 – 85.6)
A person with cardiovascular disease can safely use cannabis	65.3 (56.8 – 73.8)
Inhaled cannabis can cure asthma or COPD by its bronchodilation effect	61.3 (52.6 – 70.0)
Cannabidiol is a cannabis compound which has analgesic effects	61.3 (52.6 – 70.0)
Cannabis has anti-inflammation effects	61.3 (52.6 – 70.0)
All cannabis products are not categorized as illicit drugs	52.4 (43.5 – 61.3)
There has not been any FDA-approved cannabis product available in Thailand	41.1 (32.3 – 49.9)
Government reports are no longer required for cannabis cultivation	38.7 (30.0 – 47.4)
Concurrent use of cannabis products and chronic disease medications could result in unpredictable effects	34.7 (26.2 – 43.2)
Cannabis extracts can cure cancer	31.5 (23.2 – 39.7)

### Theme 3: Cannabis use and chronic disease self-management

#### Subtheme 1: patterns of cannabis use

*‘When I drank this (cannabis tea), I saw that my urine became clearer … After people develop diabetes, their urine becomes darker … but this (the patient’s urine) is quite clear, looked normal, and not too dark … Normally diabetic people often complain “What’s wrong with me? Why is my urine this dark?” … Yes, I instantly check the urine color. I could test when my blood sugar level was high. After a few days of drinking, I saw the color become less dark. Gotcha, it works. I used to do it that way’. (Male 52, fishery, diabetes)*

The patients used cannabis products for medical or recreational purposes or both (Table 3). The cannabis products were more likely marijuana-based than hemp-based (delta-9 THC >1% w/w, and the delta-9 THCs of the sampled cannabis extracts ranged from 0.13 to 0.95% w/w). The major reasons for medicinal use were relaxation and improving sleep quality (72.5%), and the most common preparation was cannabis tea (82.4%), of which its traditional recipe had a small amount of delta-9 THC (0.14% w/w). A quarter of the participants also used kratom (25.0%), which is a widely used plant-based psychoactive drug in southern Thailand. Two-thirds of the participants started using cannabis following the changes in cannabis regulations in 2022. A few long-term users (9.7%) started using cannabis before 2019 (median 7.5 years). Approximately half of the participants (57.3%) learned about cannabis from their friends. Most participants received cannabis from friends (49.2%) rather than from a hospital clinic (1.6%). A quarter grew their own cannabis plants, and domestic use was common, mostly in the living room (41.9%) and private room (35.5%). Some participants had used cannabis products with chronic disease medications. The prescriptions of 96 participants were accessed, and 82 of the prescriptions (85.4%) contained glipizide, simvastatin, and/or aspirin. Three participants were prescribed clopidogrel. Most participants (70.2%) from the clinic recommended cannabis to other people they knew after trying it.

**Table 3.** Patterns of cannabis use among the NCD clinic patients

Characteristic	Prevalence (95% CI)
Purpose of use	
Cure chronic disease	61.3 (52.6–70.0)
Aid sleep	56.5 (44.1–63.8)
Reduce chronic pain	16.9 (10.2–23.6)
Improve appetite	8.9 (3.8–13.9)
Medical purpose (including reduce nausea, reduce fatigue)	80.6 (73.6–87.7)
Recreation (e.g., relaxation, improve social interactions)	59.7 (50.9–68.4)
Both medical and recreation purposes	47.6 (38.7–56.5)
Type of cannabis product	
Cannabis flower or leaf tea	82.3 (75.4–89.1)
Prepared foods	20.2 (13.0–27.3)
Cannabis oil	20.2 (13.0–27.3)
Cannabis extract	15.3 (8.9–21.8)
Self-rolled cannabis cigarette	8.9 (3.8–13.9)
Timing of the recent continuous use^+^	
Before 2019	9.7 (4.4–15.0)
Between 2019 – 2022	27.4 (19.5–35.4)
After 2022	62.9 (54.3–71.5)
Frequency^#^ (past 3 months)	
Almost every day	58.1 (49.3–66.9)
Some days in a week, every week	25.0 (17.3–32.7)
A couple times per month, every month	8.9 (3.8–13.9)
A couple times in the past 3 months	8.1 (3.2–12.9)
The most common place of use	
Meeting room in the house	41.9 (33.1–50.7)
Private room	35.5 (26.9–44.0)
Friend’s accommodation	11.3 (5.6–16.9)
Restaurant	6.5 (2.1–10.8)
Workplace or other place (e.g., recreational cannabis store)	4.8 (1.0–8.7)
Risk of cannabis use^§^	
Low	13.7 (7.6–19.8)
Moderate	84.7 (78.2–91.1)
High	1.6 (0.1–3.9)
Other substance use	
Kratom (Mitragyna)	25.0 (17.3–32.7)
Cough syrup plus kratom	0.8 (0.1–2.4)
Source of cannabis products	
Friends or acquaintances	49.2 (40.3–58.1)
Self-seeking (no recommender)	36.3 (27.7–44.9)
Self-planting	22.6 (15.1–33.0)
Folk healers	10.5 (5.0–16.0)
Seniors/relatives	9.4 (4.4–15.0)
Grocery stores	3.2 (1.6–6.4)
Government hospital	1.6 (0.1–3.9)
Online retailers	1.6 (0.1–3.9)
Source of cannabis use recommendation	
Friends	57.3 (48.4–66.1)
No recommender (self-study)	38.7 (30.0–47.4)
Seniors/relatives	14.5 (8.2–20.8)
Folk doctors	13.7 (6.9–19.8)
Colleagues	4.8 (1.0–8.7)
Online resources	2.4 (0.1–5.2)
Healthcare providers	0.8 (0.1–2.4)
Ever recommended cannabis to other people	68.6 (59.5–77.8)

# modal frequency among all types of use, § assessed by ASSIST scale, +>mean (SD) = 1.9 (1.4–2.5).

#### Subtheme 2: self-management after trying cannabis

*‘My cannabis use is for medical purposes. It is unlike random self-trials. Also, I have never smoked tobacco. And these days, I rarely drink, except at certain celebrations which I was invited to join’. (Male 82, retired government officer, diabetes and rectal cancer)*

Most participants had good treatment adherence to their prescribed medications or other treatments (78.2%). Most participants maintained a healthy diet, controlling food amounts and limiting their intake of salt, sugar, and fat (57.3%); eating at least a half kilogram of vegetables and fruits daily (37.1%); and maintaining proper (feeling moderately exerted) physical activity (44.4%). More than 70% of the participants reported that they did not smoke (72.6%) or drink (75.8%). Three-quarters of the participants adopted a positive thinking approach.

### Theme 4: experienced changes after trying cannabis

*‘Three months after I tried cannabis, my doctor told me that he saw a problem in my labs. He asked what I had done because I had certain liver problems’ (Male 46, self-employment, diabetes and neuropathy)*

The perceived benefits of cannabis products varied, including acting as a sleep aid or appetite promoter to improving the quality of sexual activities. In our clinic sample, certain participants reported positive clinical changes: their doctors stepped down their medications due to better disease parameters (37.9%), some reported no change (50.0%), and a small number of self-adjustments (decreased the dosage of or stopped taking prescribed medications) (12.1%). However, negative experiences were not uncommon. Ten percent experienced hypoglycemia-like symptoms (20.7% and 4.9% glipizide-users and non-glipizide-users, respectively). Four percent of the patients reported abnormal liver function test results. All the patients who reported abnormal liver tests were currently using simvastatin, although the prevalence was higher in patients who used kratom (9.7% and 3.2% in kratom and non-kratom users, respectively).

## Discussion

This mixed-methods investigation aimed to gain an understanding of how patients with chronic disease consumed cannabis and how their self-care experience had been altered after the 2022 changes in cannabis legalization, which involved decriminalization and depenalization. Our participants were older (mean age >60 years) and mostly male. The majority had diabetes and hypertension, with a smaller proportion having cancer and cardiovascular disease. During the study period, we found that patients with chronic disease perceived easier access as both an opportunity and a threat to health. They acknowledged several health benefits and potential economic opportunities. For example, more than half of the NCD clinic participants agreed that cannabis should be promoted as a national plant. However, they were aware that cannabis consumption might impact their life and those of other people and put them at risk of undesirable effects from both the cannabinoids and unknown substances contained in poor-quality products. More than half of the participants in the second phase were concerned or uncertain about cannabis use when clinician supervision was lacking. They mostly reported using cannabis products to treat chronic diseases, although recreational use was also common. The risks of consumption were generally moderate, for which users might benefit from brief counseling before developing a cannabis use disorder (WHO ASSIST Working Group, 2002).

Many patients with chronic disease saw cannabis as having several related benefits, from symptom relief to general health promotion. Approximately 70% of our participants believed in the curative effects of cannabis for their chronic conditions. Assessing these beliefs should be a priority for health authorities, as they go beyond the scope of the Ministry of Health guidelines (‘Guidance on Cannabis for Medical Use’, 2023; ‘Guidance for Cannabis Oil (Mor Decha Formula) Under Special Access Scheme in Healthcare settings’, 2021) (i.e., insufficient scientific evidence to support medical use). The participants self-studied and mostly received recommendations regarding cannabis consumption from lay people (friends or acquaintances). These self-management and product-seeking channels have been common since the 2019 cannabis legalization (Assanangkornchai *et al.*, 2022). Additionally, we found that the majority of the usage was self-medication (only 1% consulted with physicians about cannabis use, and 2% obtained cannabis products from hospitals). We speculate that the self-medication prevalence in this relatively more liberal context was higher than that reported in a previous US national survey during the period of transition of medical cannabis law (39.6% and 18.4% in states where medical cannabis use was permitted and prohibited, respectively) (Robinson *et al.*, 2009). However, normalization of cannabis use among patients with chronic disease because of the commonness of self-medicated cannabis use should be carefully approached, as a previous study reported an increased risk of frequent withdrawal symptoms (e.g., anxiety and insomnia) from cannabis use among certain self-medicated patients (Wallis *et al.*, 2022). Moreover, cannabis use can be a ‘gateway’ to the use of other substances, especially in individuals with mental problems (Secades-Villa *et al.*, 2015). The present study found that a quarter of the participants were also using other substances (especially kratom) with their cannabis, with liver problems being more prevalent than in participants who only used cannabis. Our finding was consistent with a previous systematic review that demonstrated a potential linkage between kratom and acute liver injury (Kerrigan and Basiliere, 2022). Given such a proportion of substance co-usage and its potential risk, the history of other substance use should be queried if clinicians learn that their patients with chronic disease had used cannabis to deal with mental problems. Moreover, health authorities should publicize official cannabis clinics to ensure that the public is aware of such facilities, as a recent study from Canada reported that the lack of such knowledge was the most common reason for cannabis self-medication (52.9%) (Asselin *et al.*, 2022).

Social influence in terms of perceived cannabis-related stigma among patients was relatively low in the post-revised regulations period. Only a few participants were concerned about their care provider’s attitudes toward cannabis, and the view that cannabis did not qualify as a narcotic was prevalent. Our findings were consistent with those of a study in the United States (King *et al.*, 2024), in which the rate of cannabis disclosure in practice was considerable (57.1%), and the fear of devaluation from physicians, such as appointments being cut short, was modest. However, we speculate that patients were generally aware of some non-normalizing feedback from social contacts, as approximately half of the participants were unsure about negative reactions from their doctor if they knew about their cannabis use. Thus, it is challenging to ask patients to disclose their cannabis use and to be supervised by a health professional (Reid 2020).

Previous studies have reported that more than one-fifth of patients with diabetes and hypertension adopted CAMs for self-management (Bell *et al.*, 2006; Kretchy, Owusu-Daaku, and Danquah, 2014). Our study found that the idea of complementary use with medical prescriptions was more widely adopted by patients than as replacements. This may explain the high proportion of patients with good treatment adherence in the study sample. However, considerable risks are associated with such co-medication use. Several chronic disease medications, such as simvastatin, glipizide, and clopidogrel, could interact with cannabinoids through common paths in hepatic metabolism, and some co-uses (i.e., clopidogrel) could lead to severe side effects (MacCallum, Lo, and Boivin, 2021; ‘Cannabinoid. In: Lexicomp® Drug Interactions [database on the internet].’, no date). Therefore, to conform with the rationale of the proposed legal framework to safely promote the Thai population’s health (Sornpaisarn *et al.*, 2023), health authorities should consider preventive measures that ensure non-dangerous levels of cannabis consumption for patients with chronic disease by undertaking public awareness campaigns to educate the public and identify those inclined to use cannabis for medicinal purposes and inform them of potential dangers.

To the best of our knowledge, this is the first study to provide an in-depth understanding of patterns of cannabis use and perceptions regarding cannabis products among patients with chronic disease after the changes to non-medical cannabis use regulations in Thailand. We quantitatively described cannabis-related experiences in patients with diabetes and hypertension using a CAM approach. However, this study had several limitations. First, our participants were from a specific population of patients with chronic disease in southern Thailand. Therefore, generalizations to different populations should be performed cautiously. Nevertheless, our participants were obtained from all standard Thailand primary care settings, covering the CH, GH, RH, and MH levels; thus, their behaviors can be considered reflective of the overall national healthcare setting. Second, although we trained our data collectors to adhere to good interview manners (i.e., being attentive and non-judgmental), we could not exclude the possibility of social desirability bias, leading certain participants to rather give desirable responses to our interviewers. Lastly, this report on the impact of cannabis consumption on health was based on participants’ experiences in the past 3 months. We suggest that future studies should examine the pattern of cannabis use and its health impact over a longer period to illustrate potential long-term alterations in cannabis use behavior in patients with chronic disease.

## Conclusion

Many patients with chronic disease in the early stages of the changes in cannabis regulations perceived cannabis as both a means for improving health and potential threat to health. Some patients adopted the concepts of CAM and were influenced by the opinions of nearby acquaintances. The medicinal effects of cannabis or some legal facts were commonly misunderstood by the patients. The most common pattern was the daily consumption of cannabis tea to treat diabetes or hypertension. Certain patients with NCDs took several medications concurrently, which could result in a potentially altered treatment effect of these medications. Care providers and policymakers should consider these factors and be cautious concerning the normalization of cannabis use.

## Data Availability

The datasets used and/or analyzed during the current study are available from the corresponding author on reasonable request.
